# COVID-19: Dados Atualizados e sua Relação Com o Sistema Cardiovascular

**DOI:** 10.36660/abc.20200215

**Published:** 2020-05-22

**Authors:** Filipe Ferrari

**Affiliations:** 1 Programa de Pós-Graduação em Cardiologia e Ciências Cardiovasculares Universidade Federal do Rio Grande do Sul Hospital de Clínicas de Porto Alegre Porto AlegreRS Brasil Programa de Pós-Graduação em Cardiologia e Ciências Cardiovasculares, Universidade Federal do Rio Grande do Sul, Hospital de Clínicas de Porto Alegre, Porto Alegre, RS – Brasil

**Keywords:** Coronavirus, COVID-19, Síndrome Respiratória Aguda, Doenças Cardiovasculares/complicações, Miocardite, Doenças Infecciosas, Fatores de Risco/prevenção e controle

## Abstract

Em dezembro de 2019, um novo coronavírus humano, chamado síndrome respiratória aguda grave do coronavírus 2 (SARS-CoV-2) ou nomeado doença de coronavírus (COVID-19) pela Organização Mundial da Saúde, surgiu na cidade de Wuhan, China. Difundido globalmente, é atualmente considerado pandêmico, com aproximadamente 3 milhões de casos no mundo no final de abril. Seus sintomas incluem febre, tosse, dor de cabeça e falta de ar, esse último considerado o sintoma principal. Por sua vez, acredita-se que haja uma relação entre o COVID-19 e danos ao músculo cardíaco, e pacientes com hipertensão e diabetes, por exemplo, parecem apresentar prognóstico pior. Portanto, o COVID-19 pode piorar em indivíduos com condições adversas subjacentes. Um número não negligenciável de pacientes internados com este vírus tinham doenças cardiovasculares ou cerebrovasculares. A resposta inflamatória sistêmica e distúrbios do sistema imunológico durante a progressão da doença podem estar por trás dessa associação. Além disso, o vírus usa os receptores da enzima conversora da angiotensina (ECA), mais especificamente da ECA2, para penetrar nas células; portanto, o uso de fármacos inibidores de ECA e bloqueadores de receptores de angiotensina pode causar um aumento nestes receptores, assim facilitando a entrada do vírus na célula. No entanto, não há evidências científicas que apóiem a interrupção desses medicamentos. Considerando que são fundamentais para o manejo de certas doenças crônicas, os riscos e benefícios da sua retirada devem ser cuidadosamente ponderados neste cenário. Finalmente, cardiologistas e profissionais de saúde devem estar cientes dos riscos de infecção e se proteger o máximo possível, dormindo adequadamente e evitando longos turnos de trabalho.

## Introdução

Em dezembro 2019, na cidade de Wuhan, China, houve uma explosão de casos de pneumonia causados por um novo coronavírus, chamado síndrome respiratória aguda grave do coronavírus 2 (SARS-CoV-2),^1^ identificado como o agente que causa a doença de coronavírus (COVID-19), nome oficialmente adotado pela Organização Mundial da Saúde. O COVID-19 é uma condição que pode afetar os pulmões, o trato respiratório e outros sistemas. Dados filogenéticos sugerem uma origem zoonótica,^2^ e tem sido demonstrado que a transmissão do vírus se dá de pessoa para pessoa. Tem sido detectado em escarro, saliva, e zaragatoas da garganta e nasofaríngeas.^3^ Portanto, pode espalhar-se por meio de pequenas gotículas liberadas pelo nariz e pela boca de indivíduos infectados. Os sintomas mais observados incluem febre, fadiga, tosse seca, congestão das vias aéreas superiores, produção de escarro, mialgia/artralgia com linfopenia e tempo prolongado de protrombina.^4^ No entanto, um dos sintomas principais é falta de ar.

Embora ainda sejam pouco conhecidas as evidências sobre os efeitos específicos do COVID-19 no sistema cardiovascular, há relatos de arritmias, lesão cardíaca aguda, taquicardia e uma alta carga de doença cardiovascular concomitantes nos indivíduos infectados, particularmente naqueles com mais comorbidades e fatores de risco que necessitam cuidados mais intensivos.^5^

O diagnóstico de SARS-CoV-2 pode ser feito pela morfologia microscópica, mas o método atualmente considerado o padrão-ouro é a detecção de ácido nucléico em zaragatoa nasal, amostras da garganta ou outras amostras do trato respiratório por reação em cadeia da polimerase (RCP) em tempo real, que é subsequentemente confirmado pelo sequenciamento de próxima geração.^6^

Finalmente, é necessário observar que o melhor tratamento continua sendo a prevenção, e medidas simples, tais como lavar as mãos com sabão, utilizar álcool em gel e desinfetar superfícies como celulares, desempenham um papel essencial na redução da propagação do vírus.

### Epidemiologia

#### Adultos e Idosos

Dados mais recentes indicam que até o dia 23 de abril o número de casos confirmados de COVID-19 excediam 2.700.000 no mundo.^7^ Em 30 de janeiro de 2020, 9.976 casos de COVID-19 haviam sido relatados em pelo menos 21 países.^8^ Um mês depois, foram confirmados 83.652 casos, com 2.791 óbitos (mortalidade de 3,4%).^9^ Foram relatados casos em 24 países, em 5 continentes.^10^ No Brasil, especificamente, até 3 de março, haviam sido registrados 488 casos suspeitos, em 23 estados.^11^ Adicionalmente, até 23 de abril haviam sido confirmados aproximadamente 49.500 casos e 3.313 óbitos por COVID-19 no Brasil.^12^ Na Itália, em 20 fevereiro, um homem jovem na região de Lombardia foi internado com pneumonia atípica que mais tarde provou ser COVID-19. Durante as 24 horas seguintes, houve mais 36 casos, nenhum dos quais havia tido contato com o primeiro paciente ou com outros casos confirmados de COVID-19.^13^ Infelizmente, apesar de medidas agressivas de contenção, a doença continua a se espalhar e o número de pacientes afetados está aumentando. A taxa de letalidade não está baixa, e predomina em pacientes idosos.^12^ Portanto, atenção especial deve ser dada a essa população.

Até 23 de abril o mundo já havia registrado 2.707.356 casos de COVID-19, incluindo 83.880 casos na China. Dos 190.743 óbitos da doença até esta data, 4.636 ocorreram na China. Já a Europa tinha registrado 1.193.276 casos, com 114.259 óbitos,^7^ tornando-se a região com o maior aumento de novas infecções a cada 24 horas. Várias regiões também registraram seus primeiros casos, incluindo Somália, Benin, Libéria e Bahamas.^14^

Existem incertezas sobre as estimativas do número real de pessoas infectadas, que seria crucial para determinar a severidade da infecção e a incidência de casos leves ou assintomáticos, bem como a sua possível transmissão.^15^

#### Crianças

Fatores epidemiológicos do COVID-19 entre crianças são escassos. Por meio de análise retrospectiva de crianças com uma idade média de 7 anos incluídas no Centro Chinês para Controle e Prevenção de Doenças entre 16 janeiro e 8 fevereiro 2020, Dong et al.^16^ observaram que houve 731 casos confirmados no laboratório e 1.412 casos suspeitos. Significativamente, mais de 90% destas crianças eram assintomáticas ou apresentaram apenas sintomas leves ou moderados. Esses dados chamam atenção para o fato de que não somente adultos e idosos mas também crianças de qualquer idade são suscetíveis ao COVID-19. Por este motivo, atenção e cuidados devem ser direcionados para a população inteira, sem distinção.

## COVID-19 e o Sistema Cardiovascular

Infecções respiratórias e influenza podem desempenhar um papel importante no aumento a curto prazo do risco de infarto do miocárdio e acidente vascular cerebral isquêmico.^17^ SARS-CoV-2 possui uma patogenicidade que pode aumentar danos no miocárdio causado por esta infecção viral. Os dados sugerem que lesão cardíaca aguda, choque e arritmia estavam presentes em 7,2%, 8,7% e 16,7% dos pacientes, respectivamente, e a sua prevalência era mais alta entre pacientes que necessitaram cuidados intensivos.^10^ Baseado no fato de que o vírus pode causar danos ao sistema cardiovascular, atenção cuidadosa deve ser dada à proteção cardiovascular durante o tratamento para COVID-19.^18^ De fato, doenças cardiovasculares e hipertensão foram associadas a uma taxa de letalidade aumentada de COVID-19 na China.^19^

Lesão do miocárdio associada a SARS-CoV-2 foi relatada em 5 dos primeiros 41 pacientes diagnosticados com COVID-19 em Wuhan, os quais apresentaram níveis de troponina cardíaca I de alta sensibilidade > 28 pg/ml.^20^ Em outro estudo, realizado em 2019, Panhwar et al.^21^ observaram que infecção concomitante à influenza aumentou os riscos de pacientes internados com insuficiência cardíaca. Em uma pesquisa de 25 pacientes que haviam recuperado de infecção por SARS-CoV-1, quase a metade apresentou alterações no sistema cardiovascular, e 60% apresentou distúrbios no metabolismo da glicose.^22^ Outro estudo incluiu 1.099 pacientes com COVID-19 confirmado, dos quais 173 apresentaram doença severa, com comorbidades de hipertensão (23,7%), diabetes mellitus (16,2%), doenças coronárias (5,8%) e doença cerebrovascular (2,3%).^23^

Em uma avaliação de dados de 138 pacientes internados por COVID-19 na China, o tempo mediano entre o primeiro sintoma e a dispneia foi de 5 dias, e de 7 dias entre o primeiro sintoma e a internação hospitalar. A tomografia computadorizada do tórax mostrou sombras fragmentadas bilaterais ou opacidade de vidro fosco nos pulmões de todos os pacientes. Aproximadamente 90% dos pacientes receberam terapia antiviral com oseltamivir, e mais de 60% receberam terapia antibacteriana com moxifloxacina. Trinta e seis paciente foram transferidos para a unidade de terapia intensiva devido a complicações, incluindo síndrome do desconforto respiratório agudo (61,1%), arritmia (44,4%) e choque (30,6%). Os pacientes que necessitaram cuidados intensivos tinham idade mais avançada e maior probabilidade de apresentar comorbidades subjacentes, em adição à dispneia. Em 3 fevereiro, 34% haviam recebido alta hospitalar e 6 morreram, representando uma mortalidade geral de 4,3%.^10^

Em pacientes com COVID-19, a incidência de sintomas cardiovasculares é alta, devido à resposta inflamatória sistêmica e distúrbios do sistema imunológico durante a progressão da doença. Por este motivo, pacientes com doenças cardiovasculares subjacentes que são infectados por COVID-19 podem apresentar prognóstico pior. Atenção especial deve, portanto, ser dada à proteção cardiovascular durante o tratamento de COVID-19.

## Receptores da Enzima conversora da Angiotensina e Bloqueadores de Receptores de Angiotensina

O COVID-19 usa receptores da enzima conversora da angiotensina (ECA), mais especificamente a ECA2, para penetrar nas células. Tem sido hipotetizado que o uso de inibidores de ECA e bloqueadores de receptores de angiotensina (BRAs) possam aumentar esses receptores, assim facilitando a penetração do vírus.^24^

Em uma nota recente, a Sociedade Brasileira de Cardiologia enfatizou dados sobre a importância de tais fármacos, como inibidores de ECA e BRAs, uma vez que não há evidência clara apoiando a associação entre a terapia com esses medicamentos e prognóstico piorado da doença.^25^ Portanto, recomenda-se que os médicos cuidadosamente avaliem a relação risco-benefício antes de suspender os medicamentos, visto que são pilares fundamentais para o manejo de doenças crônicas, como hipertensão e insuficiência cardíaca. Da mesma forma, pacientes não devem interromper seu uso indiscriminadamente, sem antes consultar seus médicos.

## Considerações finais

O coronavírus é uma família de vírus que causam infecções respiratórias. O COVID-19 é uma doença grave que requer cuidados especiais. Indivíduos que apresentam febre, tosse e falta de ar devem procurar atendimento médico. Ao contrário do que muitos acreditam, o COVID-19 não é uma doença restrita aos idosos; jovens e crianças também podem ser infectados. Porém, pacientes idosos que têm doença cardiovascular infectados por COVID-19 podem apresentar prognóstico pior. Lavar as mãos frequentemente, usar álcool em gel, cobrir o nariz com a parte interna do braço e evitar ambientes aglomerados podem desempenhar um papel importante na redução da propagação do vírus e do agravamento da doença, especialmente em pacientes com doença cardiovascular.
