# Supplementary material for: COVID-19: Dados Atualizados e sua Relação Com o Sistema Cardiovascular
Source: Arq Bras Cardiol. 2020 May 22;114(5):823–6. [Article in Portuguese] doi: 10.36660/abc.20200215 (PMC8387000; doi:10.36660/abc.20200215)
Supplement: Translation

# Translation: COVID-19: Updated Data and its Relation to the Cardiovascular System

**Authors:** Filipe Ferrari

**Affiliations:** 1 Brazil Graduate Program in Cardiology and Cardiovascular Sciences, Universidade Federal do Rio Grande do Sul, Hospital de Clínicas de Porto Alegre, Porto Alegre, RS – Brazil

**Keywords:** Coronavirus, COVID 19, Acute Respiratory Syndrome, cardiovascular Diseases/complications, Myocarditis, Infectious Diseases, Risk Factors/prevention and control

## Abstract

In December 2019, a new human coronavirus, called severe acute respiratory syndrome coronavirus 2 (SARS-CoV-2) or coronavirus disease 2019 (COVID-19) by the World Health Organization, emerged in the city of Wuhan, China. Spreading globally, it is now considered pandemic, with approximately 3 million cases worldwide at the end of April. Its symptoms include fever, cough, and headache, but the main one is shortness of breath. In turn, it is believed that there is a relationship between COVID-19 and damage to the heart muscle, and hypertensive and diabetic patients, for example, seem to have worse prognosis. Therefore, COVID-19 may worsen in individuals with underlying adverse conditions, and a not negligible number of patients hospitalized with this virus had cardiovascular or cerebrovascular diseases. Systemic inflammatory response and immune system disorders during disease progression may be behind this association. In addition, the virus uses angiotensin-converting enzyme (ACE) receptors, more precisely ACE2, to penetrate the cell; therefore, the use of ACE inhibitor drugs and angiotensin receptor blockers could cause an increase in these receptors, thus facilitating the entry of the virus into the cell. There is, however, no scientific evidence to support the interruption of these drugs. Since they are fundamental for certain chronic diseases, the risk and benefit of their withdrawal in this scenario should be carefully weighed. Finally, cardiologists and health professionals should be aware of the risks of infection and protect themselves as much as possible, sleeping properly and avoiding long working hours.

## Introduction

In December 2019, in the city of Wuhan, China, there was an explosion of cases of pneumonia caused by a novel coronavirus, called severe acute respiratory syndrome coronavirus 2 (SARS-CoV-2),^1^ identified as the agent that causes the disease called coronavirus disease 2019 (COVID-19), which is the name officially adopted by the World Health Organization. COVID-19 is a condition that can affect the lungs, respiratory tract, and others systems. Phylogenetic data imply a zoonotic origin,^2^ and it has been shown that the transmission of the virus occurs from person to person. It has been detected in sputum, saliva, throat and nasopharyngeal swabs.^3^ Therefore, it can spread through small droplets released from the nose and mouth of infected individuals. Some of the most observed symptoms are fever, fatigue, dry cough, upper airway congestion, sputum production, myalgia/arthralgia with lymphopenia, and prolonged prothrombin time.^4^ However, one of the main symptoms may be shortness of breath.

Although evidence on the specific effects of COVID-19 on the cardiovascular system is still little-known, there are reports of arrhythmias, acute cardiac injury, tachycardia, and a high burden of concomitant cardiovascular disease in infected individuals, particularly in those with higher comorbidities and risk factors who require more intensive care.^5^

Diagnosis of SARS-CoV-2 can be made by electron microscopy morphology, but the method currently considered the gold standard is detection of nucleic acid in nasal swab, throat samples, or other respiratory tract samples by real time polymerase chain reaction (PCR), which is later confirmed by next generation sequencing.^6^

Finally, it should be noted that the best treatment is still prevention, and simple measures such as washing hands with soap, using alcohol gel, and disinfecting surfaces such as cell phones play an essential role in reducing the spread of the virus.

### Epidemiology

#### Adults and the Elderly

More recent data indicate that by April 23 the number of confirmed cases of COVID-19 exceeded 2,700,000 worldwide.^7^ On January 30, 2020, 9,976 cases of COVID-19 had been reported in at least 21 countries.^8^ One month later, 83,652 cases were confirmed, with 2,791 deaths (3.4% mortality).^9^ Cases were reported in 24 countries on 5 continents.^10^ In Brazil, specifically, by March 3, 488 suspected cases had been registered, in 23 states.^11^ In addition, as of April 23, approximately 49,500 cases and 3,313 deaths had been confirmed by COVID-19 in Brazil.^12^ In Italy, on February 20, a young man in the Lombardy Region was hospitalized with an atypical pneumonia that later proved to be COVID-19. In the following 24 hours, there were 36 more cases, none of which had been in contact with the first patient or anyone known to have COVID-19.^13^ Unfortunately, despite aggressive containment efforts, the disease continues to spread and the number of affected patients is increasing. The fatality rate is not low, and it is dominated by elderly patients.^12^ Therefore, special attention should also be given to this population.

By April 23, the world had already registered 2,707,356 cases of COVID-19, including 83,880 cases in China. Of the 190,743 deaths from the disease as of April 23, 4,636 occurred in China. Europe had registered 1,193,276 cases, with 114,259 deaths,^7^ making it the region with the largest 24-hour increase in new infections. Several regions have also registered their first cases, including Somalia, Benin, Liberia, and the Bahamas.^14^

There are uncertainties about the estimates of the true number of infected people, which is crucial to determine the severity of infection and the incidence of mild or asymptomatic cases, as well as their possible transmission.^15^

#### Children

Epidemiological factors of COVID-19 among children are scarce. Dong et al.^16^ through a retrospective analysis of children with a mean age of 7 years included in the Chinese Center for Disease Control and Prevention from January 16 to February 8, 2020, found that there were 731 confirmed cases in the laboratory and 1,412 suspected cases. Importantly, more than 90% of these individuals were asymptomatic or had only mild or moderate symptoms. These data draw attention to the fact that not only adults and the elderly, but also children of any age are susceptible to COVID-19. Therefore, attention and care should be directed to the entire population, without distinction.

## COVID-19 and the Cardiovascular System

Respiratory infections and influenza can play an important role in the short-term increase of risk of myocardial infarction and ischemic stroke.^17^ SARS-CoV-2 has a pathogenicity that can increase damage to the myocardium caused by this viral infection. The data suggest that acute cardiac injury, shock, and arrhythmia were present in 7.2%, 8.7%, and 16.7% of patients, respectively, and their prevalence was higher among patients requiring intensive care.^10^ Based on the fact that the virus can cause damage to the cardiovascular system, careful attention should be given to cardiovascular protection during treatment of COVID-19.^18^ In fact, cardiovascular disease and hypertension have been associated with an increased case fatality rate of COVID-19 in China.^19^

Myocardial injury associated with SARS-CoV-2 was reported in 5 of the first 41 patients diagnosed with COVID-19 in Wuhan, who had high-sensitivity cardiac troponin I levels > 28 pg/ml.^20^ In another study, conducted in 2019, Panhwar et al.^21^ observed that concomitant infection by influenza increased risks in hospitalized patients with heart failure. In a survey of 25 patients who had recovered from SARS-CoV-1 infection, almost half of them had changes in the cardiovascular system, and 60% had glucose metabolism disorders.^22^ Another study included 1,099 patients with confirmed COVID-19, 173 of whom had severe disease, with comorbidities of hypertension (23.7%), diabetes mellitus (16.2%), coronary diseases (5.8%), and cerebrovascular disease (2.3%).^23^

When evaluating data from 138 patients hospitalized for COVID-19 in China, the median time between the first symptom and dyspnea was 5 days, and 7 days between the first symptom and hospital admission. Computed tomography of the chest showed bilateral patchy shadows or ground glass opacity in the lungs of all patients. Approximately 90% of patients received antiviral therapy with oseltamivir, and more than 60% received antibacterial therapy with moxifloxacin. Thirty-six patients were transferred to the intensive care unit due to complications, including acute respiratory distress syndrome (61.1%), arrhythmia (44.4%), and shock (30.6%). Patients who required intensive care were older and more likely to have underlying comorbidities, as well as dyspnea. On February 3, 34% were discharged and 6 died, representing an overall mortality of 4.3%.^10^

In COVID-19 patients, the incidence of cardiovascular symptoms is high, due to systemic inflammatory response and immune system disorders during disease progression. Patients with underlying cardiovascular disease who are infected by COVID-19 may then have worse prognosis. Special attention should, therefore, be given to cardiovascular protection during treatment for COVID-19.

## Angiotensin-Converting Enzyme Receptors and Angiotensin Receptor Blockers

COVID-19 uses angiotensin-converting enzyme (ACE) receptors, more precisely ACE2, to penetrate the cell. It has thus been hypothesized that the use of ACE inhibitors and angiotensin receptor blockers (ARB) could increase these receptors, facilitating the penetration of the virus.^24^

In a note, the Brazilian Society of Cardiology recently emphasized data on the importance of the use of drugs such as ACE inhibitors and ARB, as there is no clear evidence to support the association between therapy with these drugs and worsening prognosis of the disease.^25^ It is thus recommended that physicians carefully evaluate the risk-benefit balance before suspending drugs, given that they are fundamental pillars for management of chronic diseases, such as hypertension and heart failure. Likewise, patients should not interrupt their use indiscriminately without first consulting their physicians.

## Final considerations

Coronavirus is a family of viruses that cause respiratory infections. COVID-19 is a serious disease, which requires special care. Individuals who present fever, cough, and shortness of breath should seek medical service. Contrary to what many may believe, COVID-19 is not a disease restricted to older adults; young people and children can also become infected. However, older patients who have cardiovascular disease and are infected with COVID-19 may have worse prognosis. Frequent hand washing, use of alcohol gel, covering the nose with the inside of the arm, and avoiding crowded environments can play an important role in reducing the spread of the virus and aggravation of the disease, especially in patients with cardiovascular disease.
